# Editorial: Human-in-the-loop cyber-physical systems

**DOI:** 10.3389/fnbot.2022.1073881

**Published:** 2022-11-14

**Authors:** Kok-Lim Alvin Yau

**Affiliations:** Department of Internet Engineering and Computer Science, Lee Kong Chian Faculty of Engineering and Science, Universiti Tunku Abdul Rahman (UTAR), Kajang, Malaysia

**Keywords:** human-in-the-loop (HITL), cyber-physical systems, artificial intelligence, augmented intelligence, smart city

This Research Topic on “*Human-in-the-loop Cyber-Physical Systems”* of Frontiers in Neurorobotics promotes outstanding research concerning human-in-the-loop cyber-physical systems (CPS), focusing on state-of-the-art progress, developments, and new trends. CPS connects our physical world and cyberspace through real-time sensing, situational awareness, and intelligent control. Approaches for processing the CPS data have evolved significantly, from the traditional manual control to autonomous intelligent decision making. With the emergence of these new approaches, the concept of CPS has evolved by integrating humans into the traditional interaction between the network and physical space. Therefore, the concept of CPHS (Cyber-Physical-Human System) has been proposed by placing people in the cycle of traditional CPS. CPHS integrates human factors, and realizes real-time, efficient and reliable data intelligence through calculation, physical interaction, human participation, and feedback loops. The core problem to achieve this goal is how to make real-time, accurate, and intelligent decisions based on the interaction of the three elements of man-machine-object and using a massive amount of sensor data.

The traditional CPS decision-making approaches are generally based on interactions between cyber and physical factors, and human factors have not been considered. The traditional approaches cannot automatically generate decision-making knowledge based on decision feedback, and historical decisions and actions. For CPHS, when we add human factors to CPS, how to formalize the relationship between different elements to guide the design of new decision-making models is the first challenge we face. Moreover, most of the existing CPS data intelligence and decision-making technologies, including sensor information fusion, time-series data management, complex event processing, etc., are dedicated to certain types of data. Some limitations include processing the single-dimensional observations of the real world, and applying them to limited application scenarios. In addition, many machine learning methods only support offline learning and require supervised training, so they cannot provide real-time, online, and unsupervised features for CPS data and decision intelligence.

While robots have been used in CPHS to integrate sensing, computation, and actuation for interactions with the physical world, significant progress must be made. So, new methods must be developed for the design, modeling, and control of robotic systems so that CPHS can work safely with humans in a CPS. In this Research Topic, seven papers were accepted for publication after rigorous peer reviews.

The article, “*Spatial-Temporal Attention Mechanism and Graph Convolutional Networks for Destination Prediction*,” by Li et al. has proposed a human-in-loop Spatial-Temporal Attention Mechanism with Graph Convolutional Network (STAGCN) model to explore the spatial-temporal dependencies for predicting destinations in traffic networks. The spatial and temporal features are identified and combined to provide a detailed input to the long-short term memory network (LSTM) to capture the spatial-temporal dependences of the traffic data. The proposed STAGCN model has shown to achieve better performance in predicting the destinations of the car hailing service.

The article, “*Research on the Dynamic Viseme of the Lip Shape based on Facial Motion Capture Technology*,” by Zhu has investigated the physiological characteristics of the lip shape during pronunciation, and has defined the facial parameter feature points of the speaker according to the MPEG-4 international standard. The investigation has shown that the distribution of lip shape changes the characteristics of different parts of the pronunciation, and the movement of the lip physiological characteristics of each speaker is random to a certain extent.

The article, “*An Augmented Neural Network for Sentiment Analysis Using Grammar*,” by Zhang et al. has proposed a novel sentimental analysis model named MoLeSy, which is an augmentation of neural networks incorporating morphological, lexical, and syntactic knowledge. MoLeSy uses convolutional neural networks (CNNs), LSTM networks, and fully connected dense neural networks. The proposed MoLeSy model has shown to achieve better performance compared to previous state-of-art models. Most importantly, morphological, lexical, and syntactic grammar can augment the neural networks for sentimental analysis.

The article, “*Application of Intelligent Inspection Robot in Coal Mine Industrial Heritage Landscape: Taking Wangshiwa Coal Mine as an Example*,” by Shen et al. has analyzed key technologies of intelligent inspection robots for industrial heritage protection in coal mines. In addition, path planning, motion obstacle avoidance, and sensing detection of robots are also studied. The investigation has shown that intelligent inspection robots have comprehensive functions to provide stable performance, which are essential to provide scientific and intelligent management approaches for industrial heritage protection.

The article, “*DeepMatch: Toward Lightweight in Point Cloud Registration*,” by Qi et al. has proposed a lightweight point cloud registration algorithm called DeepMatch. DeepMatch extracts the point features of each point, which is a spatial structure comprised of the point itself, as well as the center and farthest points of a point cloud. Because of the superiority of the point features extraction, the computing resources and time required by DeepMatch to complete the training have shown to become less than one-tenth of other learning-based algorithms. The proposed DeepMatch algorithm has also shown to reduce registration errors.

The article, “*A New Method Solving Differential Equations With Delay Based on Physics-Informed Neural Networks*,” by Feng et al. has proposed a new framework for physics-informed neural networks to solve ordinary and partial differential equations with proportional delay, subtractive delay, and time-variable delay. The well-posed system has been transformed into an optimization problem by setting a specific delay training set for the delay term in original equations. Using the auto-differentiation tools, suitable parameters enabling the neural network are identified to provide solutions with high precision. The proposed framework has showed to improve efficiency and accuracy.

The article, “*Color Constancy via Multi-Scale Region-Weighed Network Guided by Semantics*,” by Wang et al. has proposed a learning-based multi-scale region-weighed network guided by semantic features to estimate the illuminated color of light sources in a scene. Image semantics and scale information have been used to guide the process of illumination estimation. The proposed approach has shown to improve efficiency and effectiveness.

## Author contributions

The author confirms being the sole contributor of this work and has approved it for publication.

## Conflict of interest

The author declares that the research was conducted in the absence of any commercial or financial relationships that could be construed as a potential conflict of interest.

## Publisher's note

All claims expressed in this article are solely those of the authors and do not necessarily represent those of their affiliated organizations, or those of the publisher, the editors and the reviewers. Any product that may be evaluated in this article, or claim that may be made by its manufacturer, is not guaranteed or endorsed by the publisher.

